# CD43 sialoglycoprotein modulates cardiac inflammation and murine susceptibility to *Trypanosoma cruzi* infection

**DOI:** 10.1038/s41598-019-45138-7

**Published:** 2019-06-13

**Authors:** Frederico Alisson-Silva, Natália Rodrigues Mantuano, Ana Luiza Lopes, Andréia Vasconcelos-dos-Santos, André Macedo Vale, Miriam Maria Costa, Judy L. Cannon, Ana Carolina Oliveira, Adriane R. Todeschini

**Affiliations:** 1 Laboratório de Glicobiologia Estrutural e Funcional, Instituto de Microbiologia Paulo de Goes, Universidade Federal do Rio de Janeiro, Rio de Janeiro, Rio de Janeiro Brazil; 2 Laboratório de Glicobiologia Estrutural e Funcional, Instituto de Biofísica Carlos Chagas Filho, Universidade Federal do Rio de Janeiro, Rio de Janeiro, Rio de Janeiro Brazil; 3 Laboratório de Imunoreceptores e Sinalização, Instituto de Biofísica Carlos Chagas Filho, Universidade Federal do Rio de Janeiro, Rio de Janeiro, Rio de Janeiro Brazil; 4 Departamento de Bioquímica e Imunologia, Universidade Federal de Minas Gerais, Belo Horizonte, Minas Gerais Brazil; 5 Department of Molecular Genetics & Microbiology, University of New Mexico, Albuquerque, New Mexico United States of America; 6 Laboratório de Imunologia Molecular, Instituto de Biofísica Carlos Chagas Filho, Universidade Federal do Rio de Janeiro, Rio de Janeiro, Rio de Janeiro, Brazil

**Keywords:** Parasitic infection, T cells, Infection

## Abstract

CD43 (leukosialin) is a large sialoglycoprotein abundantly expressed on the surface of most cells from the hematopoietic lineage. CD43 is directly involved in the contact between cells participating in a series of events such as signaling, adherence and host parasite interactions. In this study we examined the role of CD43 in the immune response against *Trypanosoma cruzi*, the protozoan parasite that causes Chagas’ disease, a potential life-threatening illness endemic in 21 Latin American countries according to the WHO. The acute stage of infection is marked by intense parasitemia and cardiac tissue parasitism, resulting in the recruitment of inflammatory cells and acute damage to the heart tissue. We show here that *CD43*^*−/−*^ mice were more resistant to infection due to increased cytotoxicity of antigen specific CD8+ T cells and reduced inflammatory infiltration in the cardiac tissue, both contributing to lower cardiomyocyte damage. In addition, we demonstrate that the induction of acute myocarditis involves the engagement of CD43 cytoplasmic tripeptide sequence KRR to ezrin-radixin-moiesin cytoskeletal proteins. Together, our results show the participation of CD43 in different events involved in the pathogenesis of *T. cruzi* infection, contributing to a better overall understanding of the mechanisms underlying the pathogenesis of acute chagasic cardiomyopathy.

## Introduction

CD43 (Leukosialin) is a heavily glycosylated mammalian mucin-like protein expressed on the surface of most hematopoietic cells^1^. This transmembrane glycoprotein presents an extracellular domain extensively modified by *O*-linked glycans at Ser/Thr residues that extends near 45 nm from the leukocyte surface^2^ making it the most extended than all other cell-surface molecules. Because of its abundance and topographical distribution, CD43 participates in intercellular contact regulating cell adhesion and function^3^. CD43 also mediates leukocyte homing to inflammatory sites^4,5^ through binding to E-selectin expressed in endothelial cells from inflamed tissues^6^. In addition, intracellular signaling triggered by CD43 can induce T cell activation and differentiation^7,8^ and CD43 interaction with T cell receptor is important to initiate signaling events during T cell priming^9^. Additional studies revealed that CD43 function in T-cells depends on its movement to the uropod of migrating cells. The migration of the CD43 to the distal pole complex (DPC) upon T-cell activation is dependent on interaction of a tripeptide KRR sequence in the cytosolic tail of CD43 with the ERM family of cytoskeletal regulatory proteins^10^. The mutation of the KRR sequence to NGG abolishes CD43 binding to ERM proteins and, as a result, CD43 migration to the DPC is blocked^3^, which can affect T cell traffic^5,11^. Due to its high expression at the cell surface of leukocytes and its structural features, CD43 has been implicated in the immune response against pathogens such as influenza virus^12^, HIV^13^ and *Mycobacterium tuberculosis*^14^. Although Nico and collaborators have shown that CD43-expressing T cell subsets are associated with protective responses in visceral leishmaniasis^15^, little is known about the role of CD43 in the immune response against protozoan parasites.

*Trypanosoma cruzi* is the causative agent of Chagas Disease, a tropical illness that affects millions of people in Latin America^16,17^. The parasite can be transmitted to humans through the feces of contaminated insect vectors, blood transfusion, organ transplantation, laboratory accident as well as congenitally^16,18,19^. However, after interruption of vectorial transmission in South American countries^20^, the oral transmission by ingestion of food such as animal meat, vegetables and sugar cane extracts contaminated with parasite infective forms was responsible for hundreds of cases of Chagas´ disease^21,22^. Interestingly, the route of infection can differentially affect host immune response and therefore modulate disease pathogenesis^19^. The parasite infection leads to acute myocarditis and cardiomyopathy due to an intense tissue parasitism and pronounced inflammatory process, which is mainly orchestrated by T cells^23,24^. *T. cruzi* infection induces increased expression of cellular adhesion molecules such as ICAM-1, VCAM-1^25^ and E-selectin^26^ that together with other inflammatory mediators such as CCL5^27^, mediate the recruitment of T lymphocytes to the heart of infected hosts.

Previous studies from our group demonstrated that CD43 is a natural receptor for the carbohydrate binding proteins from the *T. cruzi trans*-sialidase (TcTS) family^28^. The active form of TcTS can transfer sialic acid residues from host CD43 molecules to the parasite surface glycoproteins^29^, as well as add sialic acid back to CD43 in activated CD8^+^ T cells thus modulating specific CD8^+^ T cell cytotoxicity during infection^29^. Additionally, the inactive form os TcTS (TcTS_Y342H_) acts as a lectin, binding to sialic acid in CD43 expressed in CD4^+^ T cells inducing cell proliferation and cytokine production^30^. In fact, both active and inactive isoforms of TcTS might present distinct immunomodulatory properties on T cells^31^. In this study, we used CD43 mutant mice to further exam the role of CD43 in leukocyte activation and recruitment as well as T cell function during acute cardiomyopathy induced by *T. cruzi* infection.

## Results

### CD43 mutant mice presents increased survival rate to *T. cruzi* infection

**Figure 1:**
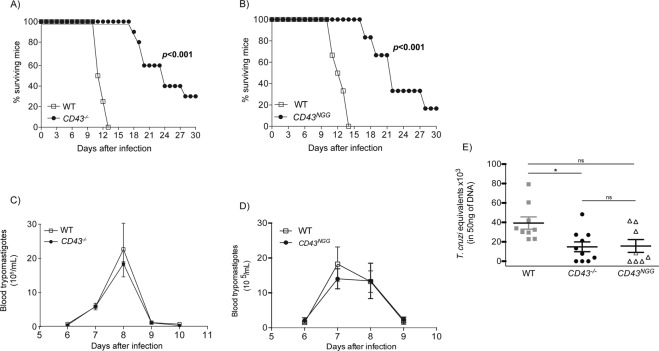
Absence or mutations in CD43 increases mice survival to infection. (**A**,**B**) Survival curve of male infected mice followed for 30 days after i.p inoculation of 10^5^ trypomastigotes (n = 10/per group *p < 0.0001). (**C**,**D**) Blood trypomastigote counts on 5uL of peripheral blood of mice from 5^th^ to the 10^th^ days after infection. Data are representative of 3 independent experiments. (**E**) qRT-PCR quantification of *T. cruzi* DNA in the heart tissue of WT (white bar) and *CD43*^*−/−*^ mice at the day 15th post infection (n = 5 each group *p = 0.0257).

To investigate the regulatory roles of CD43 during *T. cruzi* infection, we first infected *CD43*^*−/−*^ and WT mice with a lethal inoculum of 10^5^ blood trypomastigotes^32^. We also assessed infection in mice that had mutated CD43, *CD43*^*NGG*^. *CD43*^*NGG*^ mice have T cells that express a mutated form of CD43 in a key tripeptide sequence changed from KRR to NGG which was previously shown to be important for CD43 interaction with ezrin-radixin-moesin family of proteins, mediating CD43 effects on T cell migration^11^. Approximately 30% of *CD43*^*−/−*^ and 20% of *CD43*^*NGG*^ mice survived up to 30 days’ post infection while all WT mice succumbed at day 13^th^ post inoculation (Fig. 1A and B respectively). The enhanced survival rate of *CD43*^*−/−*^ and *CD43*^*NGG*^ could not be attributed to differences in the parasite blood levels since both mice showed similar levels of parasitemia from the 6^th^ to 10^th^ day post infection (Fig. 1C,D). Because the humoral response elicited against the parasite antigens is important to control the parasite spread^33^ and CD43 expression is modulated during B-1 and plasmoblast development^34,35^, we sought to analyze if the humoral response against the parasite would be affected by the absence or mutation in CD43. The analysis of serum samples from infected mice did not reveal a significant difference in either anti-*T. cruzi* IgM or total IgG levels at days 8^th^ or 15^th^ post infection (Fig. S1) suggesting that CD43 does not play a critical role in the humoral response against the parasite. Although the initial parasite replication was not affected by the lack of CD43, lower amounts of parasite DNA were found in the cardiac tissue of *CD43*^*−/−*^ and *CD43*^*NGG*^ mice when compared to WT (Fig. 1E), indicating a reduced parasite burden in the heart tissue that may explain, at least in part, the increased survival rate of both *CD43*^*−/−*^ and *CD43*^*NGG*^ mice.

### Lack of CD43 influences CD8^+^ T cell-mediated cytotoxicity and cardiac parasite load in the acute phase of infection

**Figure 2:**
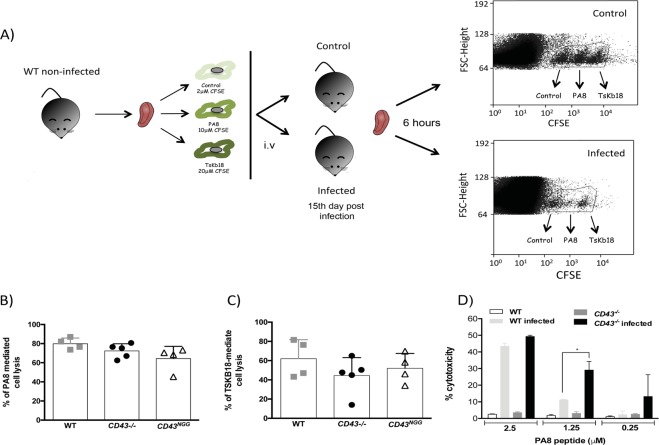
Cytotoxicity of antigen specific CD8^+^ T cells is modulated in the absence of CD43. (**A**) Experiment design to evaluate CD8^+^ T cell cytotoxicity *in vivo*. (**B**,**C**) Quantification of target cell lysis 6 hours after injection of target cells expressing PA8 or TsKB-18 peptides, respectively. (**D**) Percentage of lysis of target populations pulsed with different concentration of the CD8^+^-specific PA8 peptide 24 hours after *in vitro* incubation with total T cells isolated from infected (n = 6) or non-infected mice (n = 4). The frequency of CFSE-fluorescent cells for both *in vivo* and *in vitro* assays were determined by FACS and the percentage of lysis was determined using the formula described in the methods *p < 0.05.

Previous studies from our group demonstrated that TcTS from *T. cruzi* can alter CD43 sialylation pattern on activated CD8^+^ T cells thus reducing its cytotoxic activity^29^. Because CD8^+^ T cells are critical in the control of *T. cruzi* dissemination^36^ and host survival following acute infection^37^, and CD43 is involved in the T cells-APC contact^38^, we examined the cytotoxic activity of parasite-specific CD8^+^ T cells purified from *CD43*^*−/−*^, *CD43*^*NGG*^ and WT mice *in vivo* (Fig. 2A,B). No significant differences were found in *in vivo* CD8^+^ T cell-mediated lysis of APC’s expressing either PA8 (Fig. 2B) or TsKB18 (Fig. 2C), both CD8 specific peptides from *T. cruzi* amastigotes^39^. In contrast, the CD8^+^ T cells isolated from *CD43*^*−/−*^ infected mice displayed increased cytotoxic activity against APC’s pulsed with 1.25 μM of the immunodominant PA8 peptide *in vitro* (Fig. 2D). While CD8^+^ T cells isolated from WT induced the lysis of nearly 10% of the target population, CD8^+^ T cells from *CD43*^*−/−*^ mice induced nearly 30% of target cells lysis (Fig. 2D). Total T cells obtained from non-infected mice were used as a negative control, since no CD8^+^ T cells specific for *T. cruzi* antigens are present on naive mice (Fig. 2D). The enhanced cytotoxic activity of splenic CD8^+^ T cells from *CD43*^*−/−*^ likely contributes to the lower parasitism found in the heart tissue of the *CD43*^*−/−*^ mice.

### Differentiation of effector T cells in the spleen is compromised in the absence of CD43

**Figure 3:**
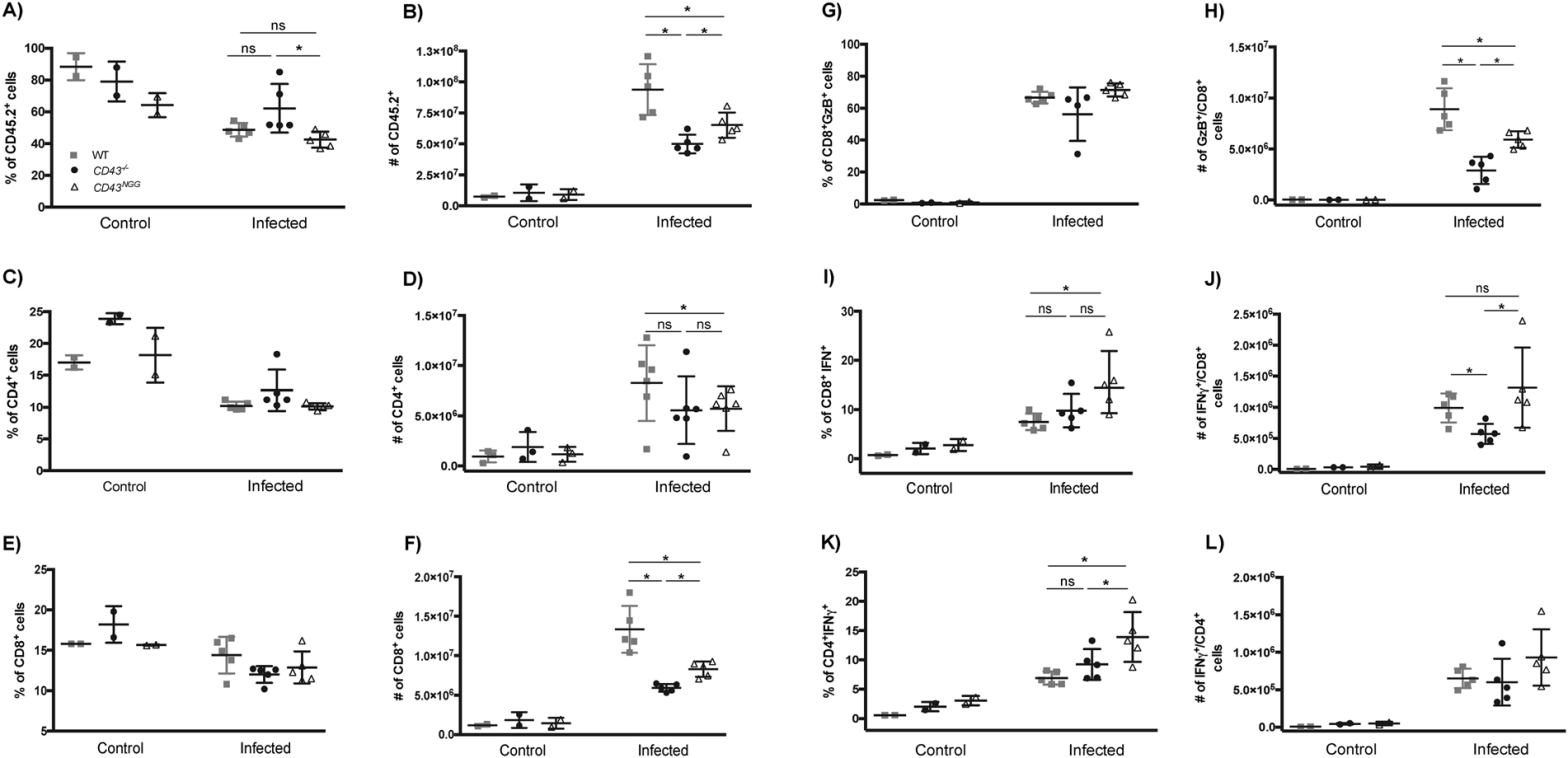
CD43 regulates the differentiation of effector T cells in the spleen. Flow cytometry analysis for the percentage and absolute number of CD45.2^+^ (**A**,**B**) CD4^+^ (**C**,**D**), CD8^+^ (**E**,**F**), CD4^+^ IFNγ^+^ (G and H), CD8^+^ IFNγ^+^ (I and J) and CD8^+^ GzB^`^ splenic T cells subsets from control (non-infected) and infected mice. Data are representative of two independent experiments. *p < 0.05. Each cell population was analysed using the gating strategy presented in Fig. S2.

Previous studies have demonstrated that CD43 acts synergistically with T cell receptor to initiate signaling events during T cell priming^9^ and that CD43 signal potentiates the expression of IFNγ by effector CD4^+^ T cells and CD8^+^ T cells^40^ therefore playing a dynamic role in the program of T cell differentiation. In keeping with this rationale, previous studies from our group have shown that TcTS interaction with CD43 induce the production of the Th_1_ cytokines IL-2 and IFN-γ^30^. Thus, we sought to investigate if the differentiation of T effector cells during *T. cruzi* infection would be compromised in the absence of CD43. In fact, either the absence of CD43 or the NGG mutation in the intracellular domain of CD43 remarkably reduced the absolute number of CD45.2^+^ cells in the spleen of infected mice (Fig. 3A,B). While the absolute number of CD4^+^ T cells was not significantly affected (Fig. 3C,D), the absolute number of CD8^+^ T cells was much lower in CD43 mutant than in WT mice (Fig. 3E,F), suggesting that CD43 signaling contributes to the CD8^+^ T cells expansion in greater extent than to the CD4^+^ T cells during infection. To further determine which effector T cell subset was mostly affected by mutations in CD43, we re-stimulated spleen cells from control and infected mice *in vitro* to perform intracellular cytokine staining. Interestingly, while both *CD43*^*−/−*^ and *CD43*^*NGG*^ mice displayed reduced numbers of GZB^+^/CD8^+^ T cells (Fig. 3G,H), only the *CD43*^*−/−*^ mice presented a significant reduction in the number of IFNγ^+^/CD8^+^ (Fig. 3I,J). On the other hand, although the percentage of CD4^+^/IFNγ^+^-producing T cells increased in *CD43*^*NGG*^ compared to *CD43*^*−/−*^and WT, there were no differences in the percentage or absolute number of CD4^+^/IFNγ^+^-producing T cells in both CD43 mutated mice compared to the WT (Fig. 3K,L). All data from Fig. 3 were analyzed by flow cytometry according to the gating strategy presented in Fig. S2. Taken together, these results demonstrate that CD43 expression and intracellular signaling directly regulates the differentiation of effector CD8^+^ T cell subsets during *T. cruz*i infection.

### CD43 is critical in the regulation of inflammatory infiltration in the heart tissue

**Figure 4:**
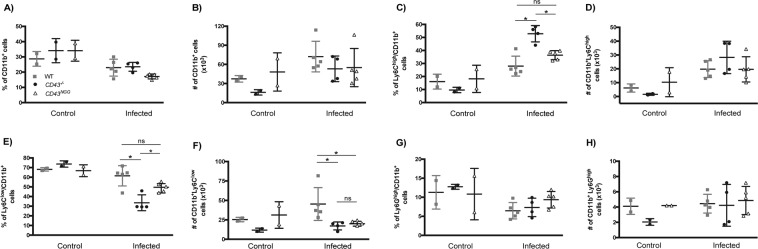
CD43 regulates the recruitment of phagocytes to the heart tissue of infected mice. Flow cytometry analysis of the frequency and absolute number of CD11b^+^ (**A,B**), Ly6C^high^/CD11b^+^ (**C**,**D**), Ly6C^low^/CD11b^+^ (**E**,**F**) and Ly6G^+^/CD11b^+^ (**G**,**H**) in the heart tissue of control (non-infected) or infected mice. Each cell population was analysed using the gating strategy presented in Fig. S3.

The heart is the most severely affected organ during the acute phase of *T. cruzi* infection^41^. Acute myocarditis is characterized by tissue parasitism and intense inflammatory infiltrate^42,43^, which can cause cardiac muscle lesions. To further examine if the altered differentiation of effector T cells would reflect in the recruitment of inflammatory cells to the cardiac tissue, the hearts of mice at the 15^th^ day post infection were harvested and digested with collagenase, in order to compare the profile of infiltrating leukocytes between WT, *CD43*^*−/−*^ and *CD43*^*NGG*^ mice by flow cytometry using the gating strategy presented in Fig. S3 (for phagocytes) and Fig. S4 (for lymphocytes). When looking for the profile of phagocytes (CD11b^+^ cells), we found an increased percentage of recruited inflammatory monocytes (CD11b^+^Ly6C^high^) in *CD43*^*−/−*^ but not in *CD43*^*NGG*^ mice when compared to WT (Fig. 4C,D). Conversely, the percentage and absolute number of CD11b^+^Ly6C^low^ was much lower in both *CD43*^*−/−*^ *and CD43*^*NGG*^ mice (Fig. 4E,F). No differences in the profile of infiltrating neutrophils (Ly6G^high^/CD11b^+^) were observed between the experimental groups (Fig. 4G,H).

**Figure 5:**
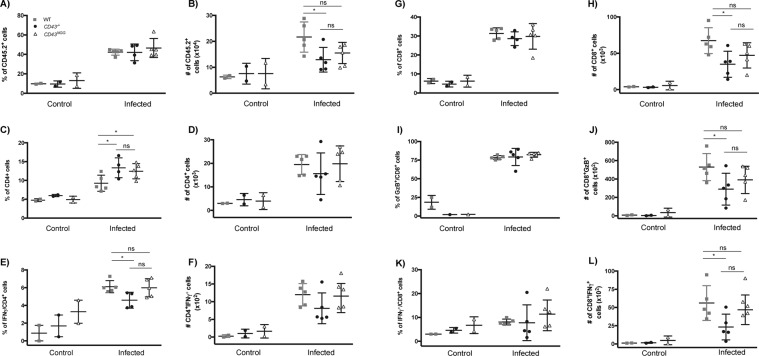
CD43 regulates migration of T cell subsets to the cardiac tissue of infected mice. Flow cytometry analyses for the relative frequencies of infiltrating T cells subsets obtained from cardiac tissue of control (non-infected) or infected mice by collagenase treatment. CD45.2^+^ (**A**,**B**), CD4^+^ (**C**,**D**), CD4^+^ IFNγ^+^ (**E**,**F**), CD8^+^ (**G,H**) CD8^+^ GzB^+^ (**I,J**) and CD8^+^ IFNγ^+^ (**K,L**). Each cell population was analysed using the gating strategy presented in Fig. S4.

Since mutations in CD43 influenced the differentiation of effector T cells subsets in the spleen (Fig. 3), we next investigated if those changes would reflect in the T cells recruitment to the cardiac tissue. Although no differences were found in the percentage of total leukocytes (CD45.2^+^) infiltrating the cardiac tissue (Fig. 5A), the absolute numbers of CD45.2^+^ cells were significantly diminished in *CD43*^*−/−*^ and *CD43*^*NGG*^ mice compared to the numbers of WT (Fig. 5B). While the reduced recruitment of IFNγ^+^/CD4^+^ T cells (Fig. 5E,F) was not enough to change the total percentage of recruited CD4^+^ T cells (Fig. 5C,D), the recruitment of CD8^+^ T cells was mostly affected by mutations in CD43 (Fig. 5G,H). Moreover, absence of CD43 impaired the recruitment of both GzB^+^- (Fig. 5I,J) and IFNγ^+^-producing effector CD8^+^ T cells (Fig. 5K,L) in *CD43*^*−/−*^ mice but the recruitment of GzB^+^/CD8^+^ T cells only (Fig. 5I,J), was reduced by the intracellular mutation in the KRR sequence present in *CD43*^*NGG*^ mice. Our data shows that in addition to its importance in the development of effector T cells in the spleen, CD43 is also critical for the recruitment of both phagocytes and effector T cells to the heart tissue.

### Acute myocarditis is drastically reduced in CD43 mutant mice

**Figure 6:**
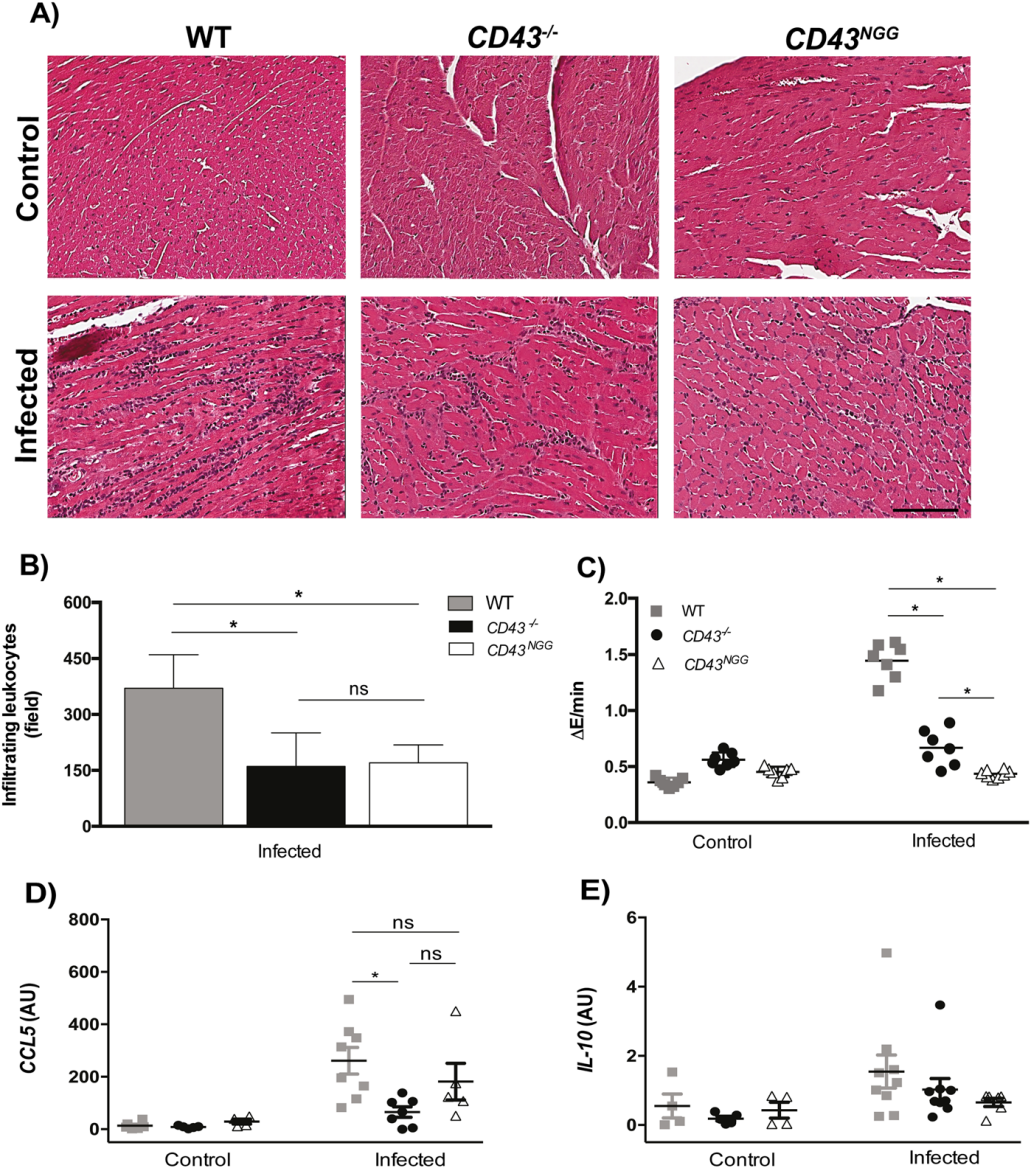
Reduced inflammatory infiltration and cardiac tissue damage in CD43 mutant mice. (**A**) Representative histology analysis of the heart tissue of control and infected mice (n = 5 mice per group). Calibration bar: 100 μm. (**B**) Quantification of heart infiltrating leukocytes in a total of 10 fields from each heart tissue sample (n = 5 mice per group - *p = 0.021). (**C**) Biochemical analysis of NAPDH levels related to the activity of the cardiac isotype of Creatine Kinase – MB. Plasma from non-infected animals was used as control for base line CK activity (*p < 0.0001). (**D**,**E**) qRT-PCR analysis for the relative expression of CCL5 (*p = 0.023) and IL-10 genes respectively.

Because the inflammatory infiltrate is directly involved in the development of the acute myocarditis, a hallmark of *T. cruzi* infection, we next performed histology and biochemical analysis to evaluate the heart tissue damage at the 15th day post infection. Corroborating the flow cytometry analysis, H&E staining revealed a smaller number of leukocytes infiltrating the heart tissue of *CD43*^*−/−*^ and *CD43*^*NGG*^ mice in comparison to that of the WT mice (Fig. 6A,B). The histology analysis also suggested that heart tissue of WT mice suffered more damage than the other mice, which we confirmed by quantifying the enzymatic activity of the cardiac isotype of creatine kinase (CK-MB) in the animals serum (Fig. 6C), an enzyme used as a marker of myocardium damage^44^. Corroborating with the reduced cardiac muscle lesion observed in CD43 mutant mice, the relative copy number of CCL5 mRNA, an important chemokine that plays a central role in the control of T cell recruitment and myocarditis development during *T. cruzi* infection^27,45^, was much lower in *CD43*^*−/−*^ than in WT mice (Fig. 6D). In addition, the reduced myocarditis could not be attributed to a lesser inflammatory environment since similar levels of IL-10, a cytokine required for the control of parasite replication as well as to prevent the development of a pathologic immune response during infection^46,47^, were found in the heart tissue of WT and CD43 mutated mice (Fig. 1E). These results indicate that CD43 plays an important role in the development of acute myocarditis by mediating T lymphocytes recruitment and CCL5 production, although not directly influencing IL-10 expression in the heart tissue during infection.

## Discussion

Our studies demonstrate that CD43 plays an important role during immune response against *T. cruzi* infection. *CD43*^*−/−*^ and *CD43*^*NGG*^ infected mice presented reduced parasite load in the cardiac tissue and impaired differentiation of effector T cell subsets that also affected the recruitment of inflammatory cells to the heart. Markedly, CD43 deficiency led to attenuated myocarditis that together with the increased CD8^+^ T cell cytotoxicity, were likely responsible for the increased survival rate of CD43 mutant mice following infection.

CD43 is a cell surface mucin-like glycoprotein that acts as physical/chemical barrier due to a highly negative charge conferred by sialic acid residues^48^. In fact, CD8^+^ T cells lacking CD43 displayed increase cytotoxicity when exposed to low concentration of viral antigens^49^. Previous studies from our lab also support the idea that sialic acid residues present on CD43 can compromise the interaction between *T. cruzi* infected APC’s and CD8^+^ T cells^29^. Activated CD8^+^ T cells express asialo glycoforms of the CD43 due to a down regulation of α2–3 and α2–6 sialyltransferases^50^. We demonstrated that the *T. cruzi* TcTS transfers sialic acid to the asialo CD43 on activated CD8^+^ T cells which decrease its cytotoxicity against the parasite^29^. Here, we show that the absence of CD43 may favor the interaction of antigen specific CD8^+^ T cell and APC’s, increasing target cell lysis and parasite killing mediated by these cells. In the *in vivo* experiment, target cells were pulsed with 2 μM of the either PA8 or TsKB18 peptides. We also did not find a significant difference in the *in vitro* cytotoxicity mediated by antigen-specific CD8+ T cells from *CD43*^*−/−*^ mice compared to cells from WT mice when using the same concentration of the PA8 (2 μM). It is important to note that *CD43*^*−/−*^ infected mice display nearly half the absolute number of spleen CD8^+^ T cells (Fig. 5F) and almost one third of the absolute number of effector GzB^+^ CD8^+^ T cells compared to the same cells from WT mice spleen (Fig. 5H). Besides this significant difference in the number of effector T cells, the *in vivo* cytotoxicity using 2uM of peptides did not show a statistical significance, which led us to conclude that CD8^+^ T cells from *CD43*^*−/−*^ are more cytotoxic than CD8^+^ T cells from WT mice. In the *in vitro* assay, we minimized the differences in cell number by enriching T cells^32^, therefore we could demonstrate the higher cytotoxicity of *CD43*^*−/−*^ CD8^+^ T cells at lower *T. cruzi* antigen concentrations. Moreover, *CD43*^*−/−*^ mice had lower parasite DNA in the cardiac tissue when compared to WT mice (Fig. 1E) showing that the absence of CD43 favored parasite killing. The reduced parasite burden in the myocardium of *CD43*^*−/−*^ mice was likely due to enhanced killing of parasites in the spleen (where the CD8+ T cell cytotoxicity was shown be enhanced in *CD43*^*−/−*^ mice) thus reducing the number of parasites that reach the cardiac tissue. The reduced parasite burden in *CD43*^*−/−*^ should not be attributed to the capacity of the parasites to invade the cardiac tissue, as cardiomyocytes do not express CD43 even in the WT mice. Together, these results point for the involvement of CD43 in the CD8^+^ T cell response against *T. cruzi*. Previous studies demonstrated that CD8^+^ T cells from *CD43*^*−/−*^ mice infected with LCMV expressed high levels of the anti-apoptotic protein B cell lymphoma 2 (Bcl-2) and thus persist longer in the tissue of mice infected^51^. It is possible that CD8^+^ T cells from *T. cruzi* infected mice persist longer in the heart tissue of *CD43*^*−/−*^, which could also favor the parasite killing. Future studies need to be done in order to elucidate this question.

Despite being widely described as ligand for E-selectin during leukocyte homing^52^, the involvement of CD43 in the recruitment of leukocytes for inflammatory sites is apparently controversial. It has been proposed that CD43 can either act as pro-adhesive molecule^4^, anti-adhesive molecule^53^ or even be not involved in leukocyte homing for inflammatory sites^54^. In keeping with this rationale, we found that absence of CD43 enhanced the recruitment of Ly6C^high^/CD11b^+^ to the heart tissue but compromised the recruitment of some effector T cells subsets (Figs 4 and 5 respectively). Analysis of infiltrating T cells revealed that the recruitment of both GzB^+^- and IFNγ^+^CD8^+^ T cells was much more affected than the recruitment of CD4^+^ T cells in the absence of CD43. The fact that activated CD8^+^ T cells express higher levels of the 130 KDa glycoform of CD43 than CD4^+^ T cells^55^ which is the glycoform of CD43 that binds to E-selectin^56^ could explain why the recruitment of the T cell subsets were differently affected by absence of CD43. The fact that CD8^+^ T cells express higher levels of CD43 glycoform that binds to E-selectin may also explain why the presence of CD8^+^ T cells is more prominent than CD4^+^ T cells in the cardiac tissue of *T. cruzi* infected mice^57^. The role of E-selectin in the recruitment of CD8^+^ T cells to the heart of *T. cruzi* infected mice can be further exploited by infection of E-selectin knockout mice.

The reduction in the inflammatory infiltrate together with lower parasite load supports the finding that *CD43*^*−/−*^ infected mice displayed reduced cardiomyopathy (Fig. 6). Lower parasite load in the cardiac tissue resulted in decreased levels of CCL5 (Fig. 6), a chemokine that is important for the recruitment of both CD4^+^ and CD8^+^ T cells effector cells to the site of infection. Thus, although apparently contradictory, our data suggest that the reduced number of IFNγ-producing CD8^+^ T cells recruited to the cardiac tissue may have resulted from both lower parasite load and reduced recruitment signals such as CCL5 in CD43^*−/−*^, on top of the role of CD43 in mediating T cell homing via engagement to E-selectin. The higher activity of the cardiac isotype of creatine kinase (CK-MB) in the serum of WT animals (Fig. 4C) was likely induced by the intense inflammatory response and parasite burden which are both reduced in the cardiac tissue of *CD43*^*−/−*^ infected mice. These results suggest that CD43-mediated recruitment of inflammatory monocytes and T lymphocytes is directly involved in the induction of acute cardiomyopathy. In fact, blocking of CD43 has been shown to reduce infiltration of T cells during inflammation of pancreatic islets and salivary glands^58^, corroborating the idea that blocking CD43 can be used as mechanism to reduce inflammation.

Our finding showing a decreased copy number of CCL5 mRNA in the heart of *CD43*^*−/−*^ than in WT mice is in line with those by Nogueira and coworkers reporting that mRNA expression of the chemokine CCL5 and its receptor was up regulated in myocardium, correlating with the intensity of the myocardial infiltrate during Chagas’ cardiomyopathy^59^. Furthermore, increased CCL5 expression in experimental infection correlates with severe cardiac pathology in beagle dogs^60^. CCL5 (RANTES) is a chemokine produced mainly by T cells, platelets, macrophages, endothelial, and epithelial cells and by the myocardial tissue of *T. cruzi*-infected mice^61^ CCL5 recruits T cells, dendritic cells, monocytes, NK cells, and other cell types^62^ to sites of inflammation and infection due to the cell surface expression of CCR1, CCR3, and/or CCR5. In agreement with our finding, treatment of *T. cruzi*-infected mice with Met-Rantes, a CCL5-based CCR5 antagonist, decreased the inflammatory heart infiltrate, with little effect on parasitism^63^.

The cytoplasmic region of CD43 is rich in serines and threonines and is highly conserved among rat, mouse, and human (>70% amino acid identity), strongly suggesting that the CD43 intracellular domain can support signal transduction. In fact, the cytoplasmic tail of CD43 regulates T cell traffic through the engagement of its tripeptide sequence KRR to ERM cytoskeleton proteins^5,11^. Here, we observed that *CD43*^*NGG*^ mice have a significant decrease in the percentage of both CD4^+^ and CD8^+^ T cells infiltrating the cardiac tissue (Fig. 5). The reduced infiltration of inflammatory T cells in *CD43*^*NGG*^ led to reduced cardiomyopathy (Fig. 6C) and increased survival rate (Fig. 6B). Together, our results suggest that the recruitment of inflammatory T lymphocytes, tissue-parasite killing and the cardiomyopathy observed during acute phase of *T. cruzi* infection are mediated by CD43 and that the tripeptide sequence in the CD43 cytoplasmic domain is required for these processes.

In both models studied here, *CD43*^*−/−*^ and *CD43*^*NGG*^, we observed an increase in the percentage of surviving mice likely due to the reduction in parasite burden and heart tissue damage. However, both mouse models studied still presented infiltration of inflammatory cells, which suggests that additional mechanisms may control the recruitment of T cells to the site of infection in the absence of CD43. It is worth noticing that while many other studies show that lack of CD43 compromises host response to infection^12,14,15^, we show that the absence of CD43 makes mice less susceptible to the pathogenesis caused by *T. cruzi* infection. In the context of Chagas’ disease, pro inflammatory responses can be detrimental as they target the parasitism in vital tissues such as the heart. Because the pathological CD43-dependent T cell responses such as the recruitment of CD8^+^ GzB^+^ producing cells (known for being involved for playing a role in the induction of myocarditis^24^) to the heart tissue is significantly reduced in *CD43*^*−/−*^ infected mice, this may explain why absence of *CD43*^*−/−*^ made mice less susceptible to *T. cruzi* infection. Thus, CD43 may either play a protective or deleterious role depending on the type of the infectious agent and mechanism of pathogenesis, therefore highlighting the importance of studying the regulatory roles of CD43 in the immune response during infection by different pathogens.

Together, our results address for the first time, the important regulatory roles of the major T cell glycoprotein CD43 in the recruitment of inflammatory cells that leads to the acute myocarditis caused by *T. cruzi* infection.

## Material and Methods

### Ethics statement

This study was carried out in strict accordance with the recommendations in the Guide for the Care and Use of Laboratory Animals of the Brazilian National Council of Animal Experimentation Control (CONCEA; https://www.sbcal.org.br/). The protocol was approved by the Committee of Ethics and Regulations of Animal use of Carlos Chagas Filho Institute of Biophysics (IBCCF214-09/16).

### Mice

*CD43*^*−/−*^ and *CD43*^*NGG*^ mice (provided by Dr. Anne I. Sperling, University of Chicago, Chicago, Illinois, USA) and C57Bl/6 were kept in a pathogen free condition at the Laboratório de Animais Transgênicos (Universidade Federal do Rio de Janeiro, Rio de Janeiro, Brazil). All experiments were conducted using mice from 8 to 12 weeks of age, according to the protocol approved by the Committee of Ethics and Regulations of Animal use of the Instituto de Biofísica Carlos Chagas Filho (IBCCF214-09/16).

### Parasites and experimental infection

Bloodstream trypomastigotes of the Y strain were obtained from *T. cruzi*-infected male C57Bl/6 mice 8 days post infection as previously described^64^. To evaluate mice susceptibility to infection, 10 males of each group received an intraperitoneal injection of 0.1 mL of phosphate buffered saline (PBS) containing 10^5^ blood trypomastigotes as previously described^64^. The injection of 10^5^ blood trypomastigotes of the Y strain is lethal for C57Bl/6 WT, inducing animal death at around 15 days post infection^32^. For all the remaining experiments, a non-lethal inoculum of 10^4^ blood trypomastigotes^32^ were injected as described above. Parasite blood levels were measured from 6^th^ to 10^th^ days post infection using Pizzi-Brenner method as previously described^65^.

### Histology analysis of heart tissue

Heart tissues were fixed in 4% buffered paraformaldehyde (PFA) for 24–48 h, dehydrated in ethanol (70%, 90% and 100%), embedded with paraffin, and 5-μm-thick sections were prepared. The slides containing tissue sections were deparaffinized in xylene and rehydrated in ethanol (100%, 90% e 70%). After rinsing 3 times with distilled water, the slides were immersed in hematoxylin for 5 min, differentiated with 1% HCl v/v in water and stained with eosin during 4 min. The slides were then dehydrated and differentiated in ethanol (70%, 90% and 100%) and mounted in entellan solution (Vector labs). Tissue sections were imaged using the digital slide scanning from Leica Biosystems (Amperio). A total of 10 fields from each heart tissue sample (n = 5 mice per group) were examined for the amount of mononuclear and polymorphonuclear infiltrating cells.

### Analysis of heart tissue lesion

Cardiomyocytes damage was assessed by measuring the activity of the cardiac isoform of Creatine Kinase (CK-MB) (Merck KGaA, Darmstadt, Germany) into 5 μL of blood plasma obtained from the tail snip of infected mice^44^. Heart tissue lesion was expressed as a rate of NADPH increase (ΔE/min) in seven sequential readings in a spectrophotometer (Molecular Devices, Sunnyvale, CA) at 340 nm. Samples from non-infected mice were used as controls for basal activity of CK-MB.

### Flow cytometry analysis of spleen cells and heart infiltrating leukocytes

Spleen cells were treated with ACK buffer for red blood cell lysis, washed twice and RPMI +10% FBS and then stained with fluorescent primary antibodies. Single cell suspensions from spleen were incubated with anti-CD16/CD32 (2.4G2 - BD) for 5 min and then stained with APC/Cy7 anti-CD45.2 (104 – Biolegend), V605 anti-CD4 (GK1.5 –Biolegend) and V421 anti-CD8a (53–6.7 – BD) for 30 min on ice. Alternatively, 2 × 10^6^ spleen cells were cultured in the presence of 5 μM of monensin (Sigma), 100 μg/mL of *T. cruzi* Y strain epimastigotes whole lysate^66^ and 10 μM of Kb-restricted PA8 peptide (VNHRFTLV) (Genscript) for 16 hr. After staining of surface markers, cells were fixed with paraformaldehyde 1% for 20 min and permeabilized with saponin 0.2% for 20 min. The permeabilized cells were then stained with either anti-granzyme B (GzB) (GB11 – Biolegend) (for CD8^+^ T cells) or PeCy7 anti-IFNγ (XMG 1.2 – Biolegend) (for CD4^+^ T cells). To analyze the profile of infiltrating-inflammatory cells in the heart tissue, the hearts from control and infected mice were sagittally sectioned, crushed and digested with 0.2% collagenase IV + DNAse (Sigma) in RPMI medium free of FBS (Sigma) at 37 °C for 60 min. Cells were washed twice in RPMI medium and all cells obtained were stained as above with anti-CD45.2, PE anti-CD11b (M1–70 - BD), BV 421 anti-Ly-6G (1A8 – Biolegend), APC anti-Ly-6C (HK1.4 – Biolegend). IFNγ-producing CD4^+^ T cells and IFNγ- or GzB-producing CD8^+^ T cells were analyzed after stimulating the cells with monensin (5 μM), 100 μg/mL *T. cruzi* Y strain epimastigotes whole lysate^66^ and 10 μM of PA8 peptide. The cells were acquired in a FACS Canto (BD) and analyzed in the FlowJo 7.10 software using the gating strategy presented in the Supplemental Figs S2–S4. To determine the absolute numbers of heart infiltrating leukocytes, all samples obtained from the heart tissue were acquired for a total time of 3 minutes and the total number of CD45.2^+^ acquired during this time was used to determine the absolute number of each leukocyte population studied.

### Cytotoxicity assay

To study the CD8^+^ T cells cytotoxicity *in vivo*, the spleen of control (non-infected) mice were harvested, macerated and ACK lysed in order to obtain enriched fractions of spleen cells. These cells were then divided in three populations that were either incubated with 2 µM of the H-2K^b^-restricted PA8 or TsKb18 peptides (ANYDFTLV)^39^ or no peptide as control, during 60 min at 37 °C in RPMI medium free of FBS. The three different populations were then stained with 2 µM (control), 10 µM (PA8) or 20 µM (TsKb18) of CFSE for the last 15 min of the total 60 min incubation time. To measure CD8^+^ T cells specific cytotoxicity, all three populations were mixed together and i.v injected in control or infected mice at the 15^th^ day post infection. Animals were euthanized and their spleen harvested and processed as described above. To study the cytotoxicity *in vitro*, enriched fractions of spleen T cells were obtained from WT mice infected (n = 6) or not (n = 4) with *T. cruzi* at 15th day after infection as previously described^32^. Total splenocytes from two uninfected mice were divided into two populations and labeled with CFSE (Molecular Probes) at 1 µM and 10 µM for 15 min at 37 °C. The population labeled with 10 µM became target after being pulsed with different concentrations (12.5, 2.5 and 1.25 mM) of the H-2Kb-restricted PA8 peptide for 40 min at 37 °C. The cells pulsed with 1.0 µM of CFSE were used as control population. All CFSE labeled populations were incubated in 24-well plates at concentration of 2 × 10^5^ cells/well (1 × 10^5^ CFSE^low^/1 × 10^5^ CFSE^high^) for 24 h in the presence of total T cells obtained from WT or *CD43*^*−/−*^ infected mice. For both *in vivo* and *in vitro* assays, the fluorescence of the target populations was detected by flow cytometry using FACScalibur (BD) and analyzed with the FlowJo 7.10 software. The percentage of specific lysis was calculated as previously described^29^.

### qRT-PCR for parasite load in the cardiac tissue

*T. cruzi* DNA in cardiac tissue from infected mice was quantified by qRT-PCR as described previously^67^. Briefly, total DNA from heart tissue of infected animals was extracted by alkaline lysis in a buffer containing 25 mM NaOH, 0.2 mM Na_2_-EDTA_2_ in H_2_O at 95 °C for 1 h. Subsequently, the material was cooled for 20 min at 4 °C and neutralized by the addition of 150 µl of a 40 mM Tris-HCl buffer. The samples were centrifuged at 8500 g for 20 min and the supernatant containing the DNA was measured by the spectrophotometer GeneQuant Calculator (Biochrom, UK) at 260 and 280 nm. The qRT-PCR reactions were performed in a 96-well MicroAmp ® Optical 96-Well Plate (Applied Biosystems, UK) and processed by the ABI Prism 7900 Sequence Detection System (Applied Biosystems, UK). The PCR conditions were: denaturation at 95 °C for 10 min, 45 cycles of 95 °C for 30 s and 60 °C for 1 min^68^. The following primer for pairs for *T. cruzi* satellite DNA– forward (5′-GCTCTTGCCCACACGGGTGC-3′), and reverse (5′-CCAAGCAGCGGATAGTTCAGG-3′) – and murine β2-microglobulin forward (5′- CTGAGCTCTGTTTTCGTCTG-3′), and reverse (5′-TATCAGTCTCAGTGGGGGTG-3′) were used.

### qRT-PCR for inflammation-related genes in the cardiac tissue

Half heart (sagittally sectioned) from control and infected mice were minced, extensively washed to avoid blood contamination and stored at −70 °C in RNA latter solution (Qiagen) until further processing. Samples were then transferred to RNAse free tubes containing TRIzol Reagent (Invitrogen, Life Technologies, USA) and homogenized with a Polytron disperser. 1ug of total mRNA were used to synthesize cDNA using the High Capacity cDNA Reverse Transcription Kit (Applied biosystems, USA) according to the manufacturer instructions.

1uL of cDNA from each sample was used to analyze the expression of *CCL5* (Forward 5′AGATCTCTGCAGCTGCCCTCA3′/Reverse 5′GGAGCACTTGCTGCTGGTGTAG-3′ and IL-10 (Forward 5′ATGGCCTTGTAG ACACCTTG-3′/Reverse 5′GCTATCGATTTCTCCCCTGTG3′) by qRT-PCR using Sybr Green Reagent (Promega, USA): Reactions were performed in the Line Gene 9600 machine (BioEr, Japan) according to the following thermal conditions: 1 step of 95 °C for 15 min, and 40 cycles of 94 °C for 15 s − 60 °C for 30 s − 72 °C for 30 s. The values of ∆CT were determined by the difference of the CT of the target gene and the CT of the housekeeping gene (β-actin). The 2^−ΔΔCT^ values were used to determine the relative copy number of each gene. All values were multiplied for one thousand and presented as arbitrary units (A.U.).

### Statistical analysis

All statistical analyses were performed using the GraphPad Prism software version 6.0. Data were compared by analyses of variance (ANOVA) followed by Tukey post-test. Relevant intervals of significance are further detailed in the figure legends. The number (N) of animals per group are displayed as scatter plot or indicated in the figure legends. Results in the figures are expressed as the mean and standard deviation (SD).

## Supplementary information

Supplementary material

## Data Availability

The datasets generated during and/or analyzed during the current study are available from the corresponding author upon request.
