# Supplementary material for: CD43 sialoglycoprotein modulates cardiac inflammation and murine susceptibility to Trypanosoma cruzi infection
Source: Sci Rep. 2019 Jun 13;9:8628. doi: 10.1038/s41598-019-45138-7 (PMC6565700; doi:10.1038/s41598-019-45138-7)
Supplement: Supplementary file 1 — Supplementary material [file 41598_2019_45138_MOESM1_ESM.pdf]

Supplementary Material

CD43 sialoglycoprotein modulates cardiac inflammation and murine susceptibility to *Trypanosoma cruzi* infection.

Frederico Alisson-Silva[1*]; Natália Rodrigues Mantuano[2], Ana Luiza Lopes[2]; Andreia Vasconcelos-dos-Santos[2]; André Macedo Vale[3]; Miriam Maria Costa[4]; Judy L. Cannon[5]; Ana Carolina Oliveira[6] and Adriane R. Todeschini[2].

Corresponding Author: frederico@micro.ufrj.br

Supplementary methods

ELISA for anti-*T. cruzi* antibodies

To determine the levels of anti-*T. cruzi* IgG and IgM in the serum of control and infected mice, ELISA was performed using anti-mouse IgG and IgM-specific reagents (Southern Biotechnology). Briefly, microplates (Half-Area, Costar, 96-well plates, cat: 3690) were coated overnight at 4°C with 100µL of 10 µg/ml of epimastigote lysates from Y strain diluted in PBS (Bermejo et al., 2011, Immunology, 132, 123-33). At the next day, the wells were washed with PBS and blocked with PBS 1% BSA (MERCK, cat: 12659) for 2 h at room temperature. Samples were diluted to 1:100, 1:300, and 1:900 in PBS-1% BSA and equal volumes of the different dilutions were incubated overnight at 4°C. At the 3rd day, the wells were washed with PBS and incubated with 100µL of either anti-mouse IgG diluted to 1:8000 or anti-mouse IgM diluted to 1:4000. Secondary antibodies were diluted in PBS-1% BSA and added for 2 h at room temperature. The wells were further washed with PBS, and the reaction revealed with OPD substrate (SIGMA- FAST P9187). The reaction was stopped with 50 µl of HCl 1N and read at 490 nm. Results are displayed as the sum of absorbance for all three dilutions.

Supplementary figures

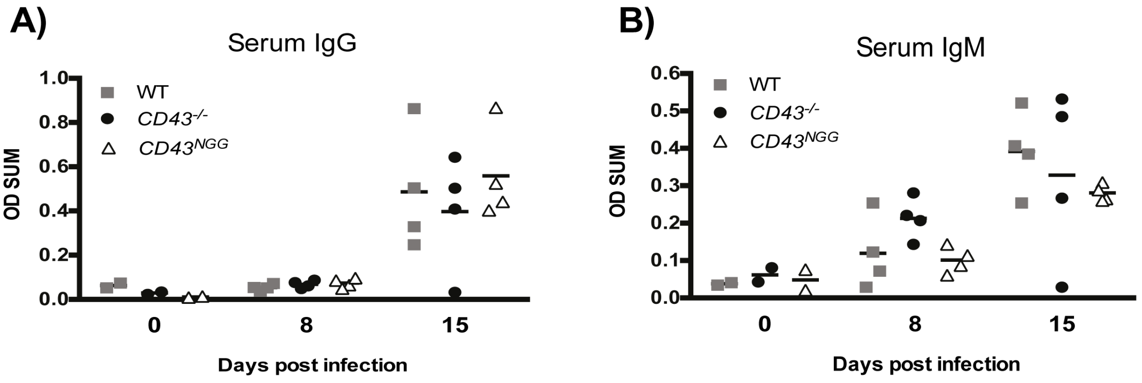

Figure S1. Complete absence or mutation in CD43 intracellular domain do not influence humoral respoe to infection. ELISA assay for the quantification of anti-*T. cruzi* IgG (A) and IgM (B) in the serum of non-infected (day 0) and infected mice at the days 8th and 15th post infection.

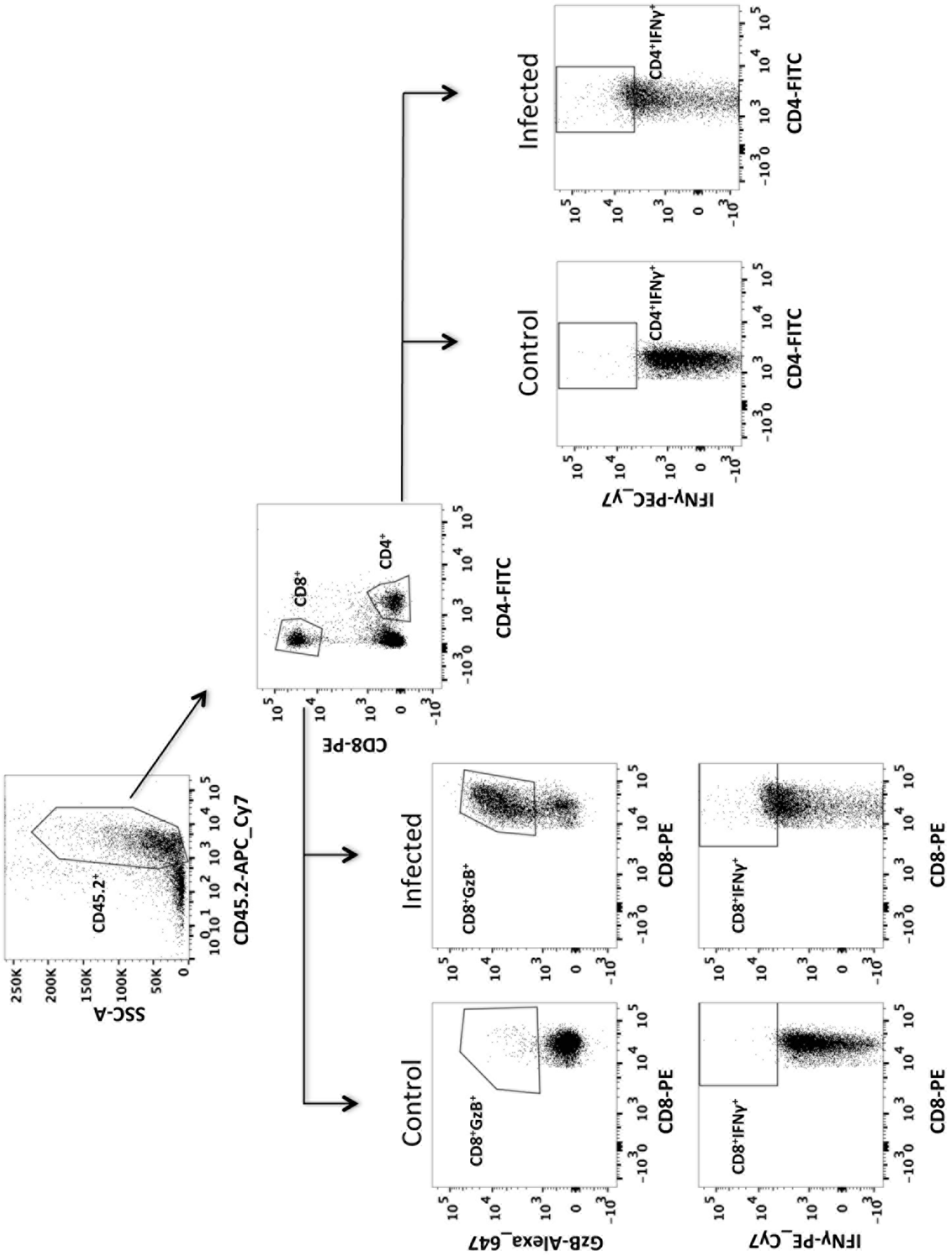

Figure S2. Gating strategy for flow cytometry analysis of spleen cells. Fluorescent antibodies used for cell surface and intracelullar staining.

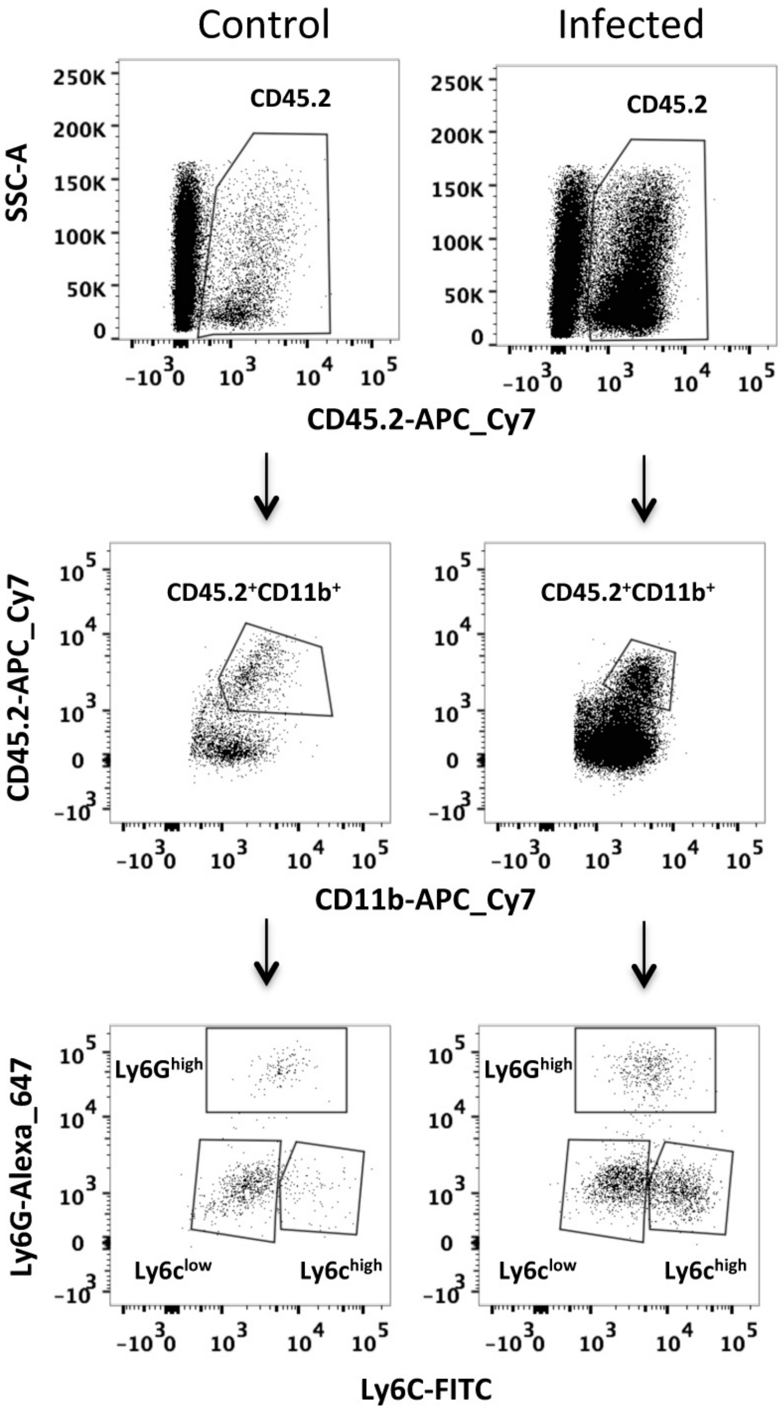

Figure S3. Gating strategy for flow cytometry analysis of heart infiltrating phagocytes. Fluorescent antibodies used for cell surface staining.

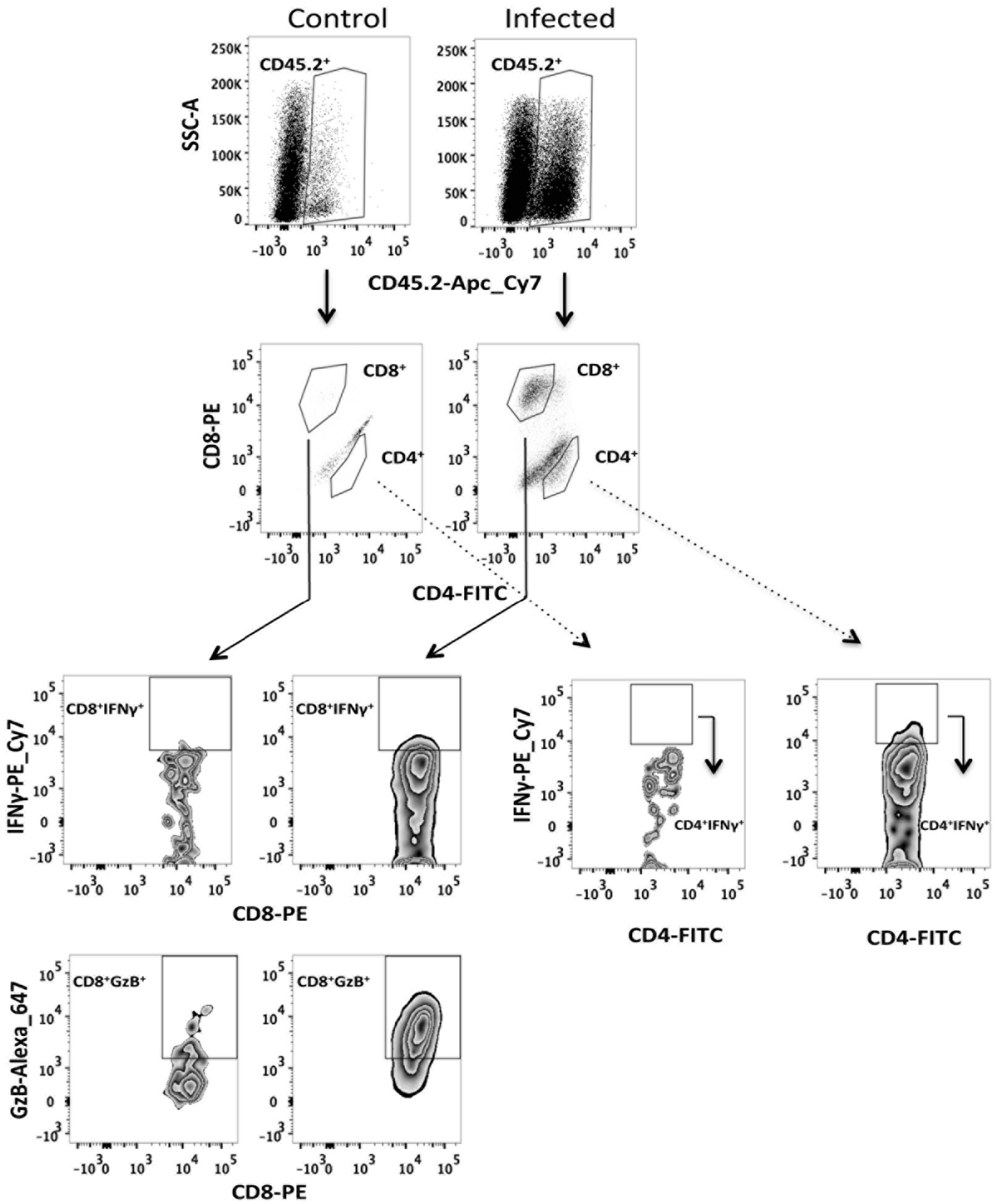

Figure S4. Gating strategy for flow cytometry analysis of heart infiltrating lymphocytes. Fluorescent antibodies used for cell surface and intracellular cytokine stainings
